# Mapping of beef, sheep and goat food systems in Nairobi — A framework for policy making and the identification of structural vulnerabilities and deficiencies

**DOI:** 10.1016/j.agsy.2016.12.005

**Published:** 2017-03

**Authors:** Pablo Alarcon, Eric M. Fèvre, Maurice K. Murungi, Patrick Muinde, James Akoko, Paula Dominguez-Salas, Stella Kiambi, Sohel Ahmed, Barbara Häsler, Jonathan Rushton

**Affiliations:** a Royal Veterinary College, London, United Kingdom; b International Livestock Research Institute, Nairobi, Kenya; c Leverhulme Centre for Integrated Research in Agriculture and Health, London, United Kingdom; d Institute for Infection and Global Health, University of Liverpool, Liverpool, United Kingdom; e University of Nairobi, Nairobi, Kenya; f University College London, London, United Kingdom

**Keywords:** Ruminant products, Food system, Nairobi, Value chain, Mapping, Markets

## Abstract

Nairobi is a large rapidly-growing city whose demand for beef, mutton and goat products is expected to double by 2030. The study aimed to map the Nairobi beef, sheep and goat systems structure and flows to identify deficiencies and vulnerabilities to shocks.

Cross-sectional data were collected through focus group discussions and interviews with people operating in Nairobi ruminant livestock and meat markets and in the large processing companies. Qualitative and quantitative data were obtained about the type of people, animals, products and value adding activities in the chains, and their structural, spatial and temporal interactions. Mapping analysis was done in three different dimensions: people and product profiling (interactions of people and products), geographical (routes of animals and products) and temporal mapping (seasonal fluctuations). The results obtained were used to identify structural deficiencies and vulnerability factors in the system.

Results for the beef food system showed that 44–55% of the city's beef supply flows through the ‘local terminal markets’, but that 54–64% of total supply is controlled by one ‘meat market’. Numerous informal chains were identified, with independent livestock and meat traders playing a pivotal role in the functionality of these systems, and where most activities are conducted with inefficient quality control and under scarce and inadequate infrastructure and organisation, generating wastage and potential food safety risks in low quality meat products. Geographical and temporal analysis showed the critical areas influencing the different markets, with larger markets increasing their market share in the low season. Large processing companies, partly integrated, operate with high quality infrastructures, but with up to 60% of their beef supply depending on similar routes as the informal markets. Only these companies were involved in value addition activities, reaching high-end markets, but also dominating the distribution of popular products, such as beef sausages, to middle and low-end market. For the small ruminant food system, 73% of the low season supply flows through a single large informal market, Kiamaiko, located in an urban informal settlement. No grading is done for these animals or the meat produced. Large companies were reported to export up to 90% of their products. Lack of traceability and control of animal production was a common feature in all chains.

The mapping presented provides a framework for policy makers and institutions to understand and design improvement plans for the Nairobi ruminant food system. The structural deficiencies and vulnerabilities identified here indicate the areas of intervention needed.

## Highlights

• Framework of interactions of the Nairobi ruminant-source food system is provided.
• Large complexity of chains, but with 64% of beef from a single meat market
• Up to 73% of sheep and goat meat sourced from informal market located in a slum
• Poor infrastructure, lack of standard grading and control of farm production
• Large companies, partly integrated, dominate value added products in the system.

## 1 Introduction

It is estimated that approximately two thirds of meat consumed in Kenya is beef. Nairobi city represents the major consumption centre for ruminant meat, with 14% of national consumption (Kenya Market Trust, 2014) and an average annual beef consumption of 15.81 kg per household in 2003 (with the lowest quintile consuming 8.55 kg) and of 19.1 kg per capita in 2014 (Gamba et al., 2005, Kenya Market Trust, 2014). In addition, the average monthly household small ruminant meat consumption was estimated at 5.5 kg in 2010 (Juma et al., 2010, Kenya Market Trust, 2014). Kenya's population of 41 million people is predicted to double and reach 97.2 million in 2050, with most of the growth concentrated in urban centres such as Nairobi (You et al., 2014). The demand for beef, mutton and goat products is predicted to double by 2030 and therefore represents a major challenge to the city (Robinson and Pozzi, 2011). Consequently, food systems will need to adapt in order to manage such a rapid increase in demand (Herrero et al., 2014). Failure to do so could have implications for food security and the achievement of dietary requirements for protein and micronutrients (Randolph et al., 2007). Despite the importance of ruminant meat products for nutrition, these are currently considered a luxury commodity for the majority of Nairobi inhabitants (Gamba et al., 2005). Access to these products is increasingly more difficult for poor consumers in informal settlements, where two thirds of the Nairobi population reside (APHRC, 2014). In addition, the way the food systems are evolving indicates an increased risk of food safety and environmental issues, with a number of well-known and manageable pathogens circulating (Kariuki et al., 2013). On the other hand, the ruminant meat sector represents an important contribution to the Kenyan economy and a major source of employment in the country and its capital (Muthee, 2006). Therefore, understanding how the food system for ruminant-based food products operates is crucial to design food policies directed at both food security and food quality, including the biological and chemical safety, which in turn contribute to sustainable economic development.

Existing information on the ruminant food system at country level indicate the main nodes, routes, gross margins and constraints (Aklilu, 2002, Alexovich et al., 2012, Bergevoet and Van Engelen, 2014, Farmer, 2012, Kenya Market Trust, 2014, Muthee, 2006). Yet there is a lack of clarity on the relative importance of formal versus informal system components, on the type of supply chains deriving from the different Nairobi markets, their control and food safety risks, among other gaps. It is also critical to consider that the ruminant food system in the city is controlled by the livestock and meat markets and large processing companies (Kenya Market Trust, 2014, Muthee, 2006). We argue that the available information on the ruminant meat food systems for Nairobi is insufficient for planning and policy purposes.

Value chain analysis is a powerful approach to assess system functionality, inefficiencies and potential opportunities for policy interventions. The first important element needed in a value chain analysis is a systematic mapping approach that takes into account people, product and chain profiles, as well as the spatial and temporal dimensions and connectivity of the system, which is essential to understand its dynamics, assess structural vulnerabilities and design effective policies (Rushton, 2009, Taylor and Rushton, 2011). It provides the critical framework needed for the investigation of chain governance, upgrading, distribution of benefits and food security risks (Hellin and Meijer, 2006, Kaplinsky and Morris, 2000, Rich and Perry, 2011, Rushton, 2009). The objective of the study presented here focuses on mapping the Nairobi beef, sheep and goat food system, in order to understand the dynamics of the system and identify existing structural deficiencies and vulnerabilities. Information generated provides a guide for policy makers for the improvement of the system. It also highlights the need for research at points in the system to ensure that the people who live and work in the system and those it feeds are given opportunities to manage their livelihoods and their nutritional needs.

## 2 Materials and methods

A cross-sectional study of the Nairobi ruminants' terminal markets, large processing companies and meat markets was conducted between February 2013 and April 2014. The research questions (RQ) were:

• RQ0 – What are the key infrastructure in the value chains – slaughterhouses, markets, input supplies?
• RQ1 - What is the structure of the different ruminant-source products chains supplying Nairobi and associated to markets and large processing companies?
• RQ2 - What proportion of the city's red meat supply is accounted for by the different chains?
• RQ3 – Who are the people directly involved in the flow of live ruminants and their products?
• RQ4 - What are the geographical routes for the supply of ruminants used by the different markets and large processing companies?
• RQ5 - What is the temporal profile of these chains?
• RQ6 - Which system deficiencies and vulnerabilities can be derived from the current structure of the chains?

### 2.1 Study area and selection of participants

**Fig. 1:**
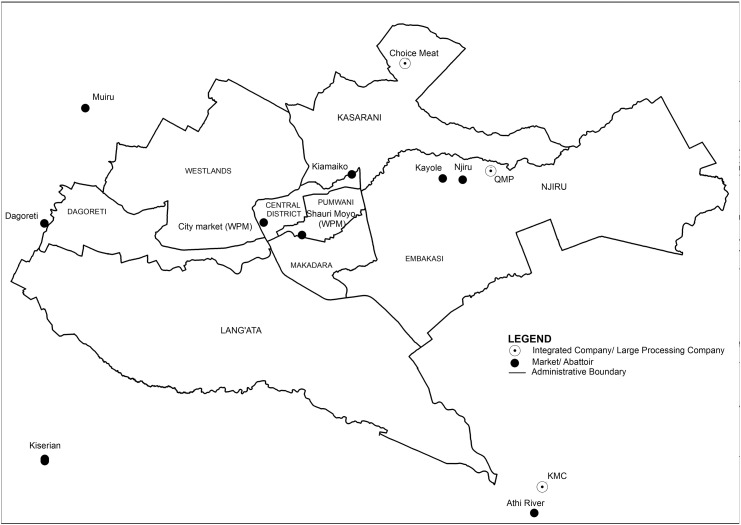
Location of principal livestock terminal markets, meat markets and large processing companies abattoirs supplying Nairobi.

Through interviews with key officers from the Ministry of Livestock Development, Department of Veterinary Services the main livestock terminal markets, wholesaler meat markets and major processing companies supplying Nairobi city were identified (RQ0) (Fig. 1). Four livestock terminal markets were visited: Dagoretti (with 4 abattoirs), Kiserian (with 2 abattoirs), Njiru (with 2 abattoirs) and Kiamaiko (with 16 abattoirs). Two meat wholesale product-only markets were also visited: Shauri Moyo and City market. The three major processing companies (each possessing their own abattoir) known to operate in the Nairobi ruminant food system were also selected for this study.

The Department of Veterinary Services authorized access to the field sites and provided introductions to the veterinary and meat inspector officers. These introduced the research team to the facility owners to obtain consent to conduct the research. An initial interview with the officers and the facility owners followed to identify and classify people in each market by their operational functions. For each operational type, 5 to 12 people were selected in collaboration with the meat inspectors or a representative of the facility owners and a focus group discussion was held. The selected people reflected diversity within each operational type (e.g. size of operation, species dealing with and other factors). Translators helped to facilitate the discussions, mostly speaking Swahili, Borana or Maasai. Where possible the presence of government officers and facility managers was discouraged to create an environment where people could share their opinions freely.

Focus group discussions were complemented with semi-structured interviews to key informants, who understood overall pattern and functionality of the market or represented a particular group of people difficult to access (such as livestock transporters). Thus, key informants were the chief veterinary officer or meat inspector of a market, a representative of the facility owner(s), or managers of the large processing companies. Other key informants were identified by these initial key informants or through discussion in the focus group discussions. In total 25 focus group discussions and 21 key informant interviews were conducted (Table 1). Where available, secondary data on animal movements were also collected. In addition, individual interviews with closed questions were conducted with nineteen abattoir managers (from different abattoirs) and six traders from Shauri Moyo market to further assess abattoir and market animal flows.

### 2.2 Data collection

In the focus group discussions participants were asked to:

(1) Briefly describe their business and operations. (RQ1 and RQ3)
(2) Identify and describe their interaction with other stakeholders. Special emphasis was placed on understanding and differentiating the diversity of suppliers, buyers and transporters of their animals or products. (RQ1 and RQ3)
(3) Identify and describe the type of animals, products and value adding activities associated to each type of people in the chain. (RQ1)
(4) Identify the routes, places, areas and seasonal differences of their interactions with the different stakeholders. (RQ4 and RQ5)
(5) Indicate the main patterns of chain flows and people existing and, when possible, to agree on the proportion of people or flow of products within a particular chain in a given market. (RQ1 and RQ2)

During the key informants' interviews participants were asked to:

(1) Describe the different types of suppliers of beef, sheep and goat animals and/or meat to the company or market and the types of operations involved with these suppliers. (RQ1 and RQ3)
(2) Describe the type of products produced by the company or market, their distribution and the type of buyers associated with each. (RQ1, RQ3 and RQ4)
(3) Provide overall annual production estimates and the proportion of flow of animals and products in the different chains. (RQ2)
(4) Describe seasonal and time patterns of the chains. (RQ5)

Data were collected using a combination of two methods: (1) the use of open ended questions (e.g. what are the different type of traders existing in the markets?); and (2) the creation of flowcharts with participants until a consensus on the type of people, products, locations, flows, and quantities, was reached. When using open questions prompts were used to further explore and clarify the activities and people, products and flows profiles. Flowcharts created with the participants were also used as a basis for formulating the open questions.

In the individual interviews to abattoir managers and traders, people were asked to indicate the high, normal and low season of trade/slaughtering using a score from 1 (low) to 3 (high) (RQ5). In addition, movement permits of animals arriving at these markets were consulted and recorded for the previous year, if available in the abattoir. Data extracted from movement permits were: (1) number of animals moved; (2) origin of transport; (3) date; and (4) species of animals moved. Data on animals slaughtered and carcasses traded for the high and low season were requested (RQ4 and RQ5).

All qualitative data from focus groups and key informants interviews were captured through video and audio recordings. Prior the focus group discussion and interviews, participants' rights (as stipulated by ILRI and RVC ethical committees) were explained and signed consent was obtained.

### 2.3 Data analysis

Through careful listening of the recordings and reading of the notes, data were collated in Word documents. Thematic content analysis of the data was done to identify the emerging themes that describe an activity or a specific profile in the chain. Templates were then used to organise these salient themes into meaningful sections (such as interaction with different stakeholders, type of suppliers, geographical factors, etc.). The templates also recorded the flowcharts obtained.

This initial process allowed to recognise major operational similarities between the food systems nodes (see Results section 3.1). These nodes were then grouped into three food system segments (‘local terminal markets’, ‘meat markets’ and the ‘large processing companies’) to facilitate the subsequent data analysis and the presentation of results. By combining data from all the templates in a segment, final data analysis of the food system was done at three levels: (1) people and product, (2) spatial or geographical and (3) temporal:

*People and product profiling* (RQ1 and RQ3) created flowcharts of the different animals, products, people and places involved in each market/company, and the movements between types of places and people. These flowcharts were combined together with the emerging themes to create system maps that indicate the chain flows, the people and products operating in a specific node. Proportion estimates were indicated where available. When disagreement was detected the source believed to be most reliable was used. To increase clarity of the diagrammatic profiles, people working in the system but not directly involved in the movement of animals and products (such as abattoir owner or regulatory officers) were omitted in the chart and listed in the narrative of these profiles. The emerging themes related to the different activities were also used in the narrative of the results to explain flows and profiles.

*Geographical mapping* (*RQ4*) identified the main physical routes for animals and carcasses to reach different markets and abattoirs through analysis of focus groups or key informant interview data or movement permits. Different origins were linked together as one route if following a similar network of roads to reach the market. Using ArcGIS Desktop platform (ESRI, Redlands, USA), the Kenya road network maps were then used to generate the different existing routes used. Movement of products within Nairobi was depicted by listing the main destination areas indicated by traders, transporters or veterinary officer of each market.

*Temporal mapping* (*RQ5*) was done by examining the contribution of markets to the Nairobi beef, sheep and goat supply for the low and the high season (RQ2). All data on animals slaughtered and carcasses traded in each market and large processing company were transformed to weekly units for comparison. Data from individual interviews with abattoir owners and traders were used to plot and compare the seasonality variations of trade in each market. For markets where sufficient movement permits were obtained, data were analysed to show seasonal variation of routes and animals traded, and the movement permits were categorised by the routes identified in the geographical mapping. The quantity traded by each route was calculated by summing the number of livestock in each of the movement permits belonging to a particular route.

Key structural system deficiencies and vulnerabilities (RQ6) were identified by the researchers through analysis of the results obtained. A deficiency was defined as factor that difficult an optimal system flows or that indicate a lack of access to some nodes in the system. A vulnerability was defined as a factor that has the potential of endangering system flows if this factor is disrupted.

### 2.4 Data validation

Initial results were presented for validation to people knowledge of the ruminant meat food systems, namely non-profitable governmental organisations, market owners, large companies' managers and veterinary officers. When data and information inconsistencies or gaps were detected, further data collection with key informants was carried out and the profiles and maps were updated.

## 3 Results

### 3.1 Food system segments and their contribution to Nairobi ruminant product supply (RQ0, RQ1 and RQ2)

Three food segment categories were created: the ‘local terminal markets’ (LTMs), the ‘meat markets’ (MMs) and ‘large processing companies’ (LPCs). The LTMs included markets such as Dagoretti, Kiserian, Njiru and Kiamaiko where:

• Live animals were sold and most were slaughtered, and their products traded.
• Operations involved many independent people with no obvious person or company dominating a significant proportion of the activities
• Clearly documented private standards and their enforcement were few. Most activities were dictated by the experience and cultural rules of independent operators, such as traders, transporters or abattoir workers.
• Carcasses were sold and traded with apparently little differentiation between different meats, albeit there were separate market flows for the offal. The value addition operations were therefore limited and trade focuses on common raw products. Products were not branded.

The MMs represented Shauri Moyo and City markets. These had similar characteristics to the LTMs, but involved movement of ruminant products only.

The LPCs represented those companies that:

• Integrated slaughtering of livestock, marketing and distribution of products, among other functions.
• Private standards (company rules) were many, and company managers carried most the responsibilities of the operations.
• Value addition of products was extensive and products are branded.

**Fig. 2:**
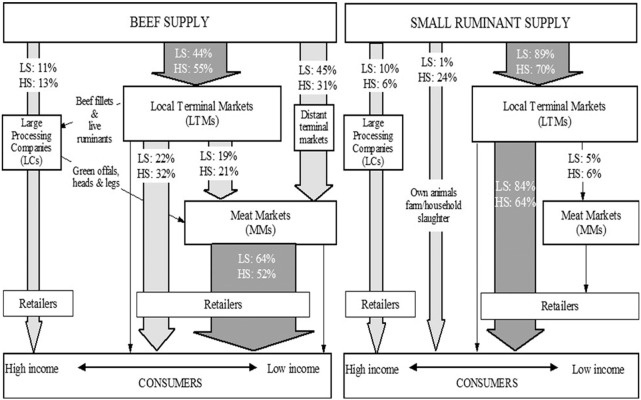
Flowchart that indicates the flow and contribution of each food segment in the supply of beef and small ruminant meat into Nairobi. The numbers in arrows indicate the percentage of all beef or small ruminant meat flows into the city for the low season (LS) and the high season (HS).

Fig. 2 shows the contribution to the city supply of ruminant meat by each food system segment, and how these interact. For beef, MMs were identified to cover up to 67% of supply to the city, and the destination point of 50% of the meat produced in LTMs. However, for the supply of small ruminant meat, the importance of MMs was minimal. Large processing companies were found to only represent 11–13% of beef meat supply and 6–10% of small ruminant meat supply.

**Fig. 3:**
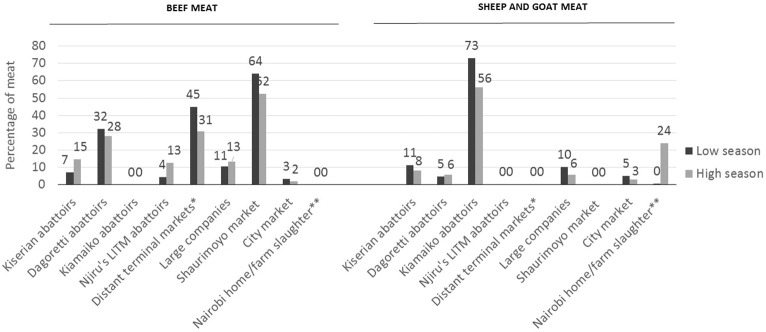
Contribution of Nairobi markets to the supply of beef, sheep and goat meat to Nairobi. *Estimation calculated based on meat arriving to Shauri Moyo and City market from other abattoirs and from Muiru abattoir in Wangige area (slaughtering 120 cows and 80 sheep and goats per week and with 25% of these distributed to Nairobi) and Athi River slaughterhouse (slaughtering 30 cows and 60 sheep and goats per week and with only 20% of these distributed to Nairobi). **Calculated based on Nairobi small ruminant population, as reported by the livestock production officers year report (2012), and assuming that one third is used for consumption in the year, and form these, half will be consumed during the high season (festive periods) with 40% slaughtered on farm (as estimated by LPOs during the focus group discussions). The other half is consumed during the low season and spread in 54 weeks. It was assumed that farm slaughter of beef in the city was insignificant.

Fig. 3 shows the contribution of each market to the supply of beef, mutton and goat meat to Nairobi city. Results showed that Shauri Moyo market (trading 2400–3000 beef carcasses per week) accounted for almost two thirds of the beef supply to the city in the low season. Dagoretti was the major live animal terminal market (slaughtering 1200–1600 cattle per week).^1^ For sheep and goats, Kiamaiko was identified as the predominant market (slaughtering 5000–10,000 small ruminants per week), accounting for almost three quarters of Nairobi supply during the low season. Estimates during high consumption periods (such as Christmas or Easter holiday) indicates that Nairobi small ruminants can represent up to a quarter of the city small ruminant supply.

Main key structural vulnerabilities identified were: (1) a large proportion of city supply was dependent on few markets, especially in the low season; (2) low income consumers were dependent on long informal chains; and (3) large companies control the high income segment.

### 3.2 People and products profiles (RQ1 and RQ3)

#### 3.2.1 Type of animals and grading systems used for beef cattle and meat

Livestock and meat traders in the LTMs reported that the best and most frequently traded cattle breed were the Boranas (price of KES 50,000 per head (USD 580^2^), followed by local breeds, such as zebu (about KES 40,000 per head (USD 464)), and Hereford and Ankole breed reported in some markets. Dairy cows (Fresian breed) were described by the meat inspectors to arrive mainly to LTMs from Nairobi and its peri-urban area.

In the LTMS and MMs no formal grading systems for live cattle were reported, but livestock traders explained that valuation is based on visual estimation of liveweight, skin coat and palpation of the back of the cattle, with fatter animals having better prices. No standard grading of beef carcasses was reported to be done either. Meat traders indicated that quality is assessed subjectively by each trader based on the perceived carcass fat content, compact/structure and source of the animal. Meat is normally differentiated into high, standard or low quality. High quality beef meat was described as “meat from an animal that is well built, not watery and that usually weighs 100–150 kgs”. However, some traders reported not to account for quality differentiation.

Two large processing companies mentioned to use specific standards for the beef cattle traded, which are linked to the quality of meat expected. Table 2 in Appendix shows the type of specifications required for beef animals for Company A. Company C however reported not to grade beef animals and meat, except on demand. Company B and C reported to mainly purchase bulls.

#### 3.2.2 Type of animals and grading systems used for small ruminants and their meat

Several participants' perceived that there is not much difference between goats and sheep. Goats however have higher prices, as their meat was believed to be preferred by consumers. Three types of sheep were described to be traded in LTMs: Red Maasai, Doper and the Black headed Persian. No formal grading of small ruminants and their meat was reported to be done, and similar informal criteria as for beef were used.

Large integrated processing companies stated to mainly require young goats weighing between 4 and 10 kg for export. Any goat outside this category was said to be used for local Kenyan market and sold at a cheaper price. For sheep LPCs' customers were reported to prefer Merino and Doper breed.

#### 3.2.3 Local terminal markets (LTMs) profile for beef cattle and products

**Fig. 4:**
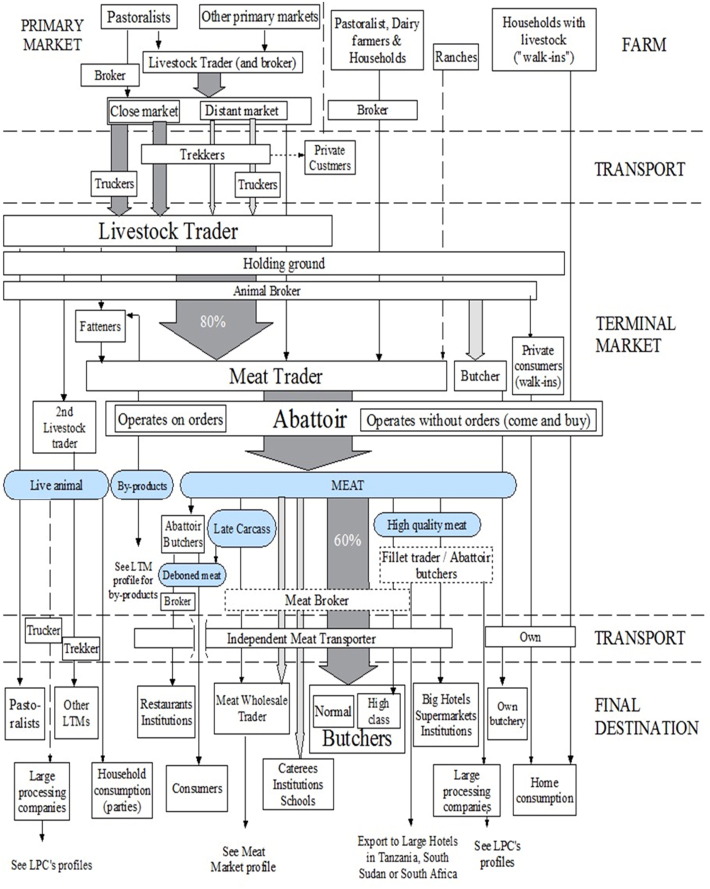
People and product profile of the ‘local terminal markets’ operating in Nairobi. Footnote: Circles indicate commodities traded, arrows indicate the flows of products, dotted arrow indicate rare flows, boxes indicate people or places, and dotted boxes indicate occasional flow through. Late carcass refers to carcasses exposed for long hours or several days and that have suffered decolouration.

Fig. 4 shows the results of the people and product chain profile for the LTMs.

##### 3.2.3.1 Source of cattle

Primary markets were identified by traders as the most common source of animals and livestock traders (those that buy and sell live cattle) were perceived to be the main suppliers of cattle into LTM. Meat traders (those that sell meat) stated to only occasionally do this on times of shortages. Traders described that in the primary markets animals are bought most frequently through brokers and that cattle could be bought and sold in up to three primary markets before reaching the terminal market, with the price of animals increasing in each transaction. Some livestock were also reported by these traders to come from other LTMs in Nairobi to profit from higher market capacity for those animals difficult to sell. Occasionally, some nearby pastoralists were mentioned to bring livestock directly to these markets because of higher prices. Dairy farmers in the urban and peri-urban area were also seen to use this route to sell their old cows for replacement and frequently do it through brokers in their area. The use of large ranches, with up to 2000 head of cattle, was described as a rare source, in decline and only practiced by large and established meat traders.

**Fig. 10:**
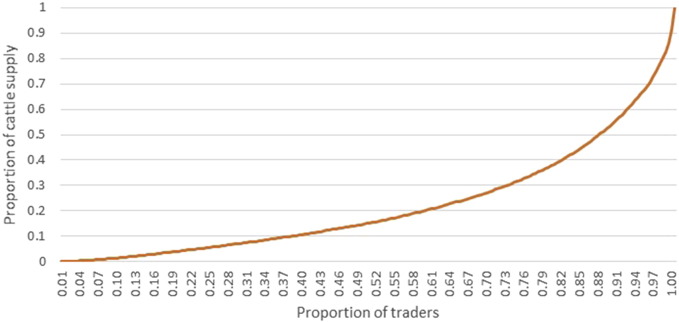
Lorenz curve showing the proportion of supply related to different proportion of traders in Dagoretti abattoirs for the month of March and December.

Analysis of movement permits for cattle in Dagoretti and Njiru abattoirs showed that the average trader transports 19.4 and 22.9 cattle per movement permit, respectively. Only few traders (four traders in Dagoretti and 13 traders in Njiru) were observed to bring > 30 cattle. However, of the 200 traders analysed through the Dagoretti movement permits for the month of December 2012 and March 2013, only 12% accounted for the supply of 50% of cattle to this market (Fig. 10 in Appendix).

##### 3.2.3.2 Transport of cattle

Transport of cattle from the primary market was described to be organised by traders. In Kiserian and Dagoretti most animals from nearby markets, from the same district as the terminal market or from distant south areas were reported to be trekked. Trekking of cattle from the border of Tanzania to Kiserian (5 days trek from ‘Shompole market’) and from Narok to Dagoretti (2 days trek) were identified as frequent. Trekkers were described to transport about 100 animals at once (between beef, sheep and goats), belonging to 8–10 different livestock traders. These stated to sell between 3 and 10 animals, on behalf of the trader, to customers along the way, and also to collect animals from other traders. Cattle from north distant areas and those going to Njiru abattoirs were reported to be trucked. A truck was estimated to transport about 18–23 cattle from 3 to 5 different traders twice per week, depending on the distance of origin. These trucks were perceived to be owned by independent people, who could possess from 1 to 6 trucks (only one large company was reported to exist, which owns about 23 trucks). These trucks, after delivering the animals, are often used to transport people back to rural areas. In Kiserian, trucking was reported to be done mainly when a trader has an urgent need of animals.

##### 3.2.3.3 Transactions of live cattle within the markets

In the major LTMs (Dagoretti and Kiserian), it was described that once the animal arrive they are put into a ‘holding ground’. For other LTMS, such as those situated in Njiru area, cattle are put in a pastoral area near the abattoirs. It is in the ‘holding ground’ (or pastoral area) where the transactions were reported to occur. Meat traders estimated that 80% of the cattle in the terminal markets are bought by them from livestock traders. However, livestock were identified to be sold also directly to some butchers who prefer to buy animals instead of carcasses. Some weak or young animals were said to sometimes be bought by other traders for fattening for about three months in places as far as the Maasai Mara region or in ‘Manyattas’ (Maasai cattle holding structures) in the peri-urban areas of Nairobi.

Traders described that cattle transactions in LTM markets were frequently done through brokers, who operate in two different ways. Either they purchase animals on credits and sell them at a higher price, but doing both transactions in the market on the same day (more frequent in times of shortages), or traders/pastoralists offer the broker a fee for finding clients who can buy or sell animals at a certain price. Livestock traders reported also to operate often as brokers, which represented about 10% of their business activities.

Although most cattle were said to be sold a few days after arrival, some were reported to remain in the market (at the holding ground) for up to 1.5 or 2 weeks until they are slaughtered or move to another terminal market. During this period animals are moved to the road and nearby fields or forests for grazing and watering. For the Njiru markets and one large company, cattle can remain in the pastoral areas near the abattoirs for up to 1 month. About 700 to 800 animals were estimated to arrive per week to this area. During a peak month up to 2000 animals, mostly cattle, were estimated to be kept in these areas.

##### 3.2.3.4 Slaughtering and transaction of beef products

In LTMs the meat trader was identified as the main person who organises the slaughtering of animals and the selling of products. Meat traders were differentiated in two types: large and small traders. Large meat traders, believed to be the majority, have the capacity to buy 8 to 10 beef animals (and up to 20–30) per day, while small traders buy < 8 (between 1 and 2 cattle generally) beef animals per day.

Abattoirs in these LTMS were reported to operate in two ways, which reflects how carcasses are sold:

• Mainly without order: Traders slaughter animals without having a client. Carcasses are hung in the clean area of the abattoir where clients are sought, in 10%–50% of cases with the help of meat brokers. This practice was reported in the majority of LTMs abattoirs.
• With orders: In these meat traders will have an existing selling agreement with a client before slaughtering the animals.

##### 3.2.3.5 Beef meat chains

Most ‘standard quality carcasses’ were reported to be sold to “normal” butchers, representing the destination of 60% of meat in these markets. Higher quality meat was perceived to be more demanded by butchers in Nairobi, and standard meat to be demanded by butchers in the outskirt of the city. Meat traders also reported to sell standard quality carcasses to other meat traders operating in MMs (second in ranking for Dagoretti meat traders), to institutions and schools (third in ranking) or to caterers. Carcasses identified as ‘high quality’ were reported to be sold mainly to “high class” butchers. In some abattoirs, ‘fillet traders’, who buy special muscle parts of the cows from butchers buying carcasses, were identified. These reported to sell fillets to 4 or 5 star hotels, institutions (hospitals, schools, airport, Non-governmental and governmental institutions), or to export them to other countries' hotels (in Tanzania, South Sudan and South Africa), supermarkets or to some “high class” butcheries. Large processing companies were also reported to buy fillets from Dagoretti and Njiru's markets in period of shortages.

In most abattoirs that operate without orders, it was explained that some carcasses remain hung and unsold at the end of the day (named in Fig. 4 as ‘late carcasses’) due to their low quality or because cattle were slaughtered too early (affected by preferences for fresh meat by clients) or too late on the day, when customers are few. Many of these carcasses were said to be sold for a cheaper price to traders that operates in MMs or to be deboned and sold to small restaurants and consumers. Traders reported that a carcass could remain unsold up to three days until these are disposed. Deboning is also done with average quality meat and commonly sold to restaurants, institutions and consumers.

##### 3.2.3.6 Beef offal, heads and legs chains

**Fig. 5:**
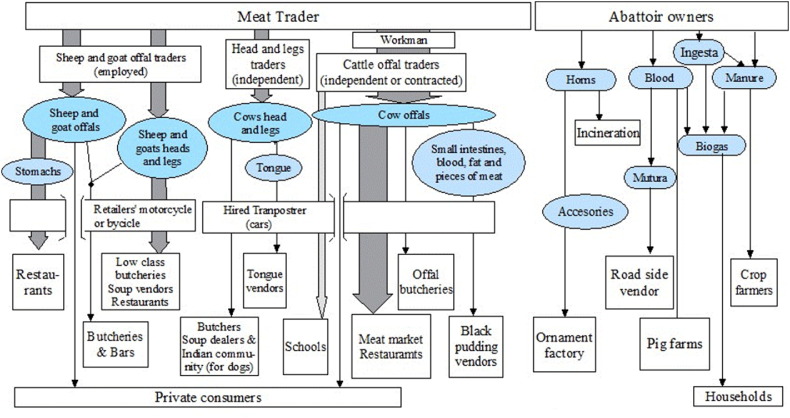
Profile of by-products trade by the local terminal markets operating in Nairobi. Footnote: Circles indicate commodities traded, arrows indicate the flows of products and their width indicate importance in terms of flow. Mutura is the Swahili word for black pudding.

Offal traders described to be generally specialized either on small ruminant or on beef offal. For beef offal, the meat traders stated to employ a workman who, among other duties, sells the cow offal, heads and legs to offal traders and head traders. Distribution of these products are shown in Fig. 5. In some markets it was reported that most offal, heads and legs are sold to retailers in informal settlements areas.

##### 3.2.3.7 Product transportation

The majority of meat and offal transportation was reported to be done by hiring independent transporters who mainly use hired motorcycles and small trucks/cars with meat boxes. Transporters and traders described that trucks are most frequently used to transport meat to the Central Business District, the big hotels or large processing companies. They have the capacity to transport up to 10 beef carcasses, while motorcycles can only transport up to 1.5 beef carcasses or 200 kg of meat. Beef offal was reported to be mostly transported in cars with meat boxes, due to the large quantities sold and their heavy weight. Meat and offal were said to be transported separately in different containers. Only liver is transported with the meat, wrapped in polythene paper. Meat traders and butchers were identified as the people that organise transport of products. The meat transporters were reported to be separated in groups within a market, each with a route or region to supply.

The structural deficiencies in beef LTMs (RQ6) identified related to:

• Weaknesses of the processes in the system: lack of standardised grading; lack of product differentiation or value addition processing activities; and slaughtering without orders and carcasses sold in abattoir clean areas.
• Poor linkage to production and transport systems: lack of access to ranches and to high quality animals; long trekking of animals; and lack of system traceability.
• Overall minimal investments in cold chain: long stay of animals in holding ground and movement of these around the city; and several carcasses unsold at the end of the day and move to another market or sold to low class restaurants or low income consumers.

The structural vulnerabilities in beef LTMs (RQ6) identified indicated reliance on transaction systems that concentrate power to few people: numerous transaction for animals and extensive broker activity; livestock and meat traders controlling 80% supply of animals and meat; few livestock traders controlling 50% of supply; and 60% of all meat distributed to butcheries.

#### 3.2.4 Local terminal markets (LTMs) profile for small ruminant and their product

The chains and operations for sheep and goats were similar to the beef system, but with the following main differences observed:

• Source of sheep and goats: During high season, festive periods, or for party purposes, sheep and goat were also reported to come into the LTM as “walk-ins” from (1) households, mainly urban and peri-urban, that own a few sheep and/or goats, and (2) households without livestock who purchase a goat or sheep in the LTM holding ground. These households slaughter the small ruminant at the market abattoir and take it home for consumption. These walk-ins were estimated to represent up to 70% of animals slaughtered during these festive days. It was explained that these do not respect the 24 h quarantine in the lairage of the abattoirs.
• Transport of sheep and goats: These animals were described to be mostly trucked, as they are more susceptible to fatigue. A truck was estimated to transport about 150–200 sheep or goats. Some trekking activity was reported in Kiserian (maximum period trek of 2 days), or from nearby farms.
• Slaughtering and distribution of sheep and goat meat products: In Kiamaiko, animal brokers, instead of herders, were identified as the people responsible for the feeding and watering of small ruminants. All abattoirs in Kiamaiko were reported to operate without orders and without lairage. Sheep and goat carcasses were described to be sold together with their heads, flanks, kidneys and liver to high class and standard butcheries and to bars. High quality small ruminant meat were reported to be normally sold to butchers rather than to supermarkets, while fillets are rarely obtained from these animals.
• Sheep offal, heads and legs: Sheep and goat offal and heads were reported to be most frequently sold to retailers by the meat traders directly, due to their small size and their small value. Restaurants were perceived to represent the large majority of the clients for offal, but were described to only buy the stomachs, as their customers do not like the intestines. In Kiamaiko bars and black pudding vendors were reported to be the main clients for intestines. Sheep and goat heads were indicated to be mostly sold to small “low class” retailers that operate in the area next to market or in Nairobi informal settlements.
• Product transport: In Kiamaiko, transporters were categorised mainly in two groups: those who are able to transport 20–30 sheep or goat carcasses in a day and those who only are able to transport 1–2 sheep or goat carcass in a day. Bicycles with meat boxes were also identified and described to be used for short distances and to frequently transport sheep and goat offal. In Kiamaiko, it was estimated that 95% of offal transport is organised by the retailers themselves. Only large transporters were believed to own their vehicles.

Main structural deficiencies identified were the same as for beef in LTMs, with the addition of (1) lack of access to supermarkets, (2) lack access to fillet traders and (3) lack of control of animals in lairage. Main vulnerabilities were similar to those described in beef, with the addition of the importance of ‘walk-ins’ representing 70% supply in key festive days.

#### 3.2.5 Meat markets (MMs) profile for beef meat

**Fig. 6:**
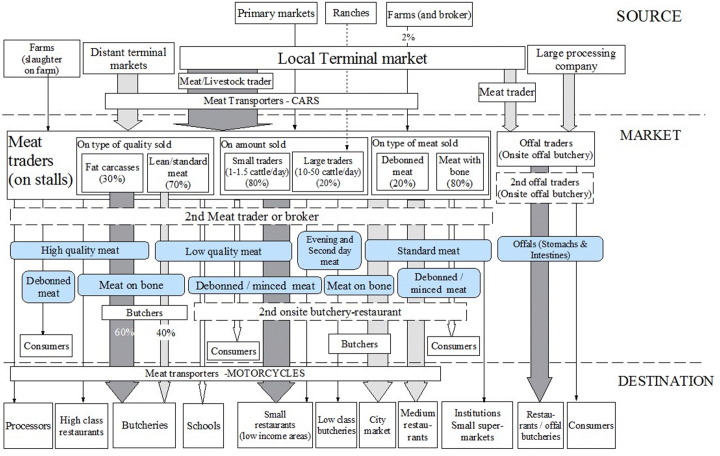
People and product profile for Shauri Moyo market. Footnote: Circles indicate commodities traded, arrows indicate the flows of products, dotted arrow indicate rare flows, boxes indicate people or places, and dotted boxes indicate occasional flow through. Percentage shown in meat trader box correspond to percentage of traders in each category.

The profile corresponding to Shauri Moyo market is shown in Fig. 6.

In this market meat was described to be brought by meat traders who have stalls to display carcasses. These traders were classified in different ways based on: selling meat on bone (80%) or deboned meat (20%); selling fat (30%) or lean (70%) carcasses; the quantity sold, with large and small traders, and the type of registration, with those belonging to registered companies (15%) or those operating as individuals (85%). The majority of traders (80%) in Shauri Moyo market sell small quantities (1–1.5 carcasses/day) of lean beef meat on bone and operate as registered individuals. Meat traders in this market were also classified as 1) ‘wholesale meat traders’, who are traders bringing carcasses into the market, having stalls and selling large quantities to all businesses; and 2) ‘meat retailers’, who have onsite butcheries selling small quantities particularly to consumers and restaurants outside the market. In total 27 wholesale traders (5 selling high quality meat, 15 selling low quality and 7 selling standard meat) and about 10 meat retailers were reported to operate in Shauri Moyo. In City market, most trader have butcheries and sell on average 2 beef carcasses per day, but with one trader was estimated to account for 40% of the supply.

##### 3.2.5.1 Source of products

The majority of the meat was reportedly purchased in nearby LTMs by wholesale traders in the market. On the other hand, about 90% meat retailers were estimated to purchase their meat from the wholesale traders inside the market, except in periods of shortages when they can source their meat directly from Nairobi LTMs. Occasionally meat originates from animals slaughtered on farms, with the inspection done at the market gate. Onsite restaurants reported to buy meat mainly from the market meat retailers. Offal, stomachs and intestine butcheries were abundant in Shauri Moyo market and explained to buy their products from meat traders operating in LTMs and LPCs. For City market, 40% of beef meat was reported to be source from Shauri Moyo market, 25% from Limuru abattoir, 10% from Dagoretti market, 5% from Kayole market and 5% from Kiambu slaughterhouse.

##### 3.2.5.2 Beef meat distribution

Butchers were reported to be the main purchasers of bone meat, with the majority buying high quality. Deboned standard meat was indicated to be mainly sold to butcheries in City market or to medium class restaurants, and to some institutions and small supermarkets. “High class” restaurants (representing 5% of all restaurants supplied) were mentioned to require high quality meat on bone from Boran cattle. However, these and small supermarkets were reported to obtain their beef meat from butcheries at City market and rarely from Shauri Moyo market. Meat traders described that deboned and minced low quality meat was sold predominantly to small restaurants situated in low income areas and to a lesser degree to schools. Meat that stays unsold overnight or for two days was indicated to also be sold to small restaurants in informal settlements and to low class butchers who come in the evening hours to benefit from cheaper meat prices. About 70% of butchers and restaurants coming to Shauri Moyo market were estimated to buy the meat through meat brokers, who operate as a representative of the meat trader. Several private consumers were also reported to purchase and/or consume any type of meat quality in this market.

In City market, 35% of meat was estimated to be sold to medium and large restaurants and bars, 30% to institutions (government, schools and hospitals), 15% to consumers, 10% to large restaurants, 10% to small supermarkets, 5% to snack shops and 5% to others. Small restaurants were reported to not purchase meat in this market.

##### 3.2.5.3 Transport of beef meat

Transporters explained that most large traders own transport vehicles to carry carcasses from various abattoirs to Shauri Moyo, while small traders were reported to use the transport from these big traders for a fee. Transport from abattoirs to Shauri Moyo was described to be done by cars, while motorcycles are used for transports from the market to butcheries and restaurants.

The structural deficiencies identified were: lack of access to institutions and supermarkets by Shauri Moyo and lack of access to small restaurant by City market; lack of product differentiation or value addition processing activities; long supply chains (especially with meat moving to second MM), meat overstay and sold to low income retailers or consumers (food safety risks); and few registered companies. The structural vulnerabilities identified were: extensive brokering activity; transport controlled by large meat traders; butcheries principal customers of fat meat; small restaurant main customers of the market; and important dependency to LTMs.

#### 3.2.6 Meat markets (MMs) profile for small ruminant meat

City market was identified as the only MM that sells small ruminant carcasses, with two thirds of sales being goat and one third sheep carcasses. All small ruminant meat was reported to originate from LTMs, 90% from Kiamaiko market. The meat is distributed as follows: 30% to institutions, 25% to medium and large restaurants, 10% to bars, 15% to supermarkets, 10% to consumers and 10% to other retailers such as snack shops.

#### 3.2.7 Large processing integrated companies profiles for beef meat

**Fig. 7:**
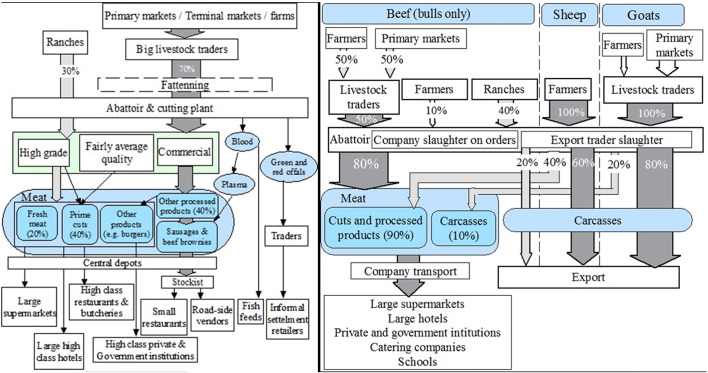
People and product profile for three large processing companies Fig. 7 People and product profile for three large processing companies Footnote: Circles indicate commodities traded, arrows indicate the flows of products, dotted arrow indicate rare flows, boxes indicate people or places, and dotted boxes indicate occasional flow through. Percentage shown in meat trader box correspond to percentage of traders in each category.

Fig. 7 show the profiles for the three large processing companies.

##### 3.2.7.1 Source of cattle

Large processing companies reported to operate in a similar manner to the LTMs for the supply of beef, with some differences. Over half of the beef cattle supply was done by independent livestock traders, who sourced their animals from primary markets and, occasionally, from Nairobi terminal markets. Company B required livestock traders to fatten the animals for some months before arrival at the abattoir. Company A on the other hand reported to purchase beef in large quantities during the livestock abundance period (dry season), when prices are cheaper, and to keep them in a buffer zone (ranch) next to the abattoir. The buffer animals were described to be used during periods of shortages to help the company meet demand. Cattle brought to this buffer were reported to be 2–3 years old and to stay in it for 12 months. Beef cattle from livestock traders provide commercial, standard and some fair average quality grade carcasses. Livestock traders supplying these companies were required to operate with large quantities (for value of 20 million KES or about 40 cattle). One company reported that the minimum purchase quantity allowed was 20 beef cattle. On the other hand, it was explained that few traders use the large processing companies' abattoirs just for slaughtering services and mainly for export purpose. For the two companies, these were estimated to represent up to 20% or 60% of the beef slaughtered. Large companies also reported to obtain beef cattle from ranches and these were indicated to represent the main source for the highest quality meat.

##### 3.2.7.2 Cattle transportation and slaughtering

It was reported to be organised by traders and ranchers, who hire trucks. No holding ground or market activity of animals was mentioned to exist near their premises of the companies. Animals arriving are kept overnight in the lairage (with water ad-lib), weighed and slaughtered the following day. Grading was indicated to be done by specialist graders, on cattle carcasses only. Cattle carcasses were explained to be kept in chillers for 5–7 days to enhance natural ageing. These chillers have capacity of 370 cattle carcasses and up to 1000 small ruminant carcasses.

##### 3.2.7.3 Value addition

These companies reported to perform extensive value addition activities for the beef products. Main value added products are sausages, meat balls, meat burgers, prime cuts and canned products. The inedible by-products are: hooves, horns, skin & hides and masks. The edible by-products processed are mainly meat, bone and blood meal. Proportions obtained from each type of quality meat are shown in the different profiles. Products were described to be packaged and labelled with the company's name.

##### 3.2.7.4 Distribution of beef products

Company A estimated that about 60% of beef meat (mainly canned beef) is sold to government and private institutions. The rest is traded to high end customers, and with one quarter of beef meat supplied to large supermarkets; mainly the prime cuts and value added products. A small proportion of meat was indicated to be sold to butcheries, restaurants, schools and consumers. Some meat and bone meals were reported to be sold to dog owners and for pig and poultry feed. For company B, fresh meat marketed was described to derive mostly from high grade carcasses, while processed meat is obtained from commercial grades. For the fresh meat and prime cuts, the main customers were indicated to be the large supermarkets, large hotels (5 and 4 stars), high end butcheries, high end restaurants and high end private and government institutions. The processed meat, mainly sausages, were sold to the mass market through central depots that in turns distributes to several stockists located throughout Nairobi. These sells to small restaurants and road-side vendors (especially trolley vendors). Between 80 and 100 tonnes of beef sausages were reported to be sold per week, representing 65% to 84% of company's products. For company C, the main destination of products was indicated to be the large supermarkets, large hotels (5 and 4 stars), private and government institutions (such as hospitals), catering companies, schools and few butcheries. Export of beef was reported by company A and C and in small proportions, mainly to Middle East countries.

##### 3.2.7.5 Transport of beef meat

This was described to be integrated by the companies that own refrigerated vehicles (vans and trucks) and employ the transporters.

Main deficiencies identified were: Lack of traceability and control on farm production; and lack of access to middle-income or low income customers (with the exception of sausages distribution). Main structural vulnerabilities identified were: Small market niche, dependent on high income retailers, institutions and tourism; and supply dependency on same primary market as used by the LTMs, with dependency on livestock traders (limited direct supply from farm/ranges).

#### 3.2.8 Large processing integrated companies profiles for small ruminant meat

Only Company A and C reported to purchase and sell small ruminants and associated products. The main difference observed with beef cattle was that all goats in both companies and most sheep in Company A were sourced from livestock traders, who obtained their animals from primary markets. However, company C sourced all their supply of sheep directly from farms or ranches. Most of small ruminant carcasses were reported to be exported after overnight stay in chillers and only heavier animals are used for local markets. Company C sold sheep in the form of special cuts or processed products, while goats are only marketed in the form of carcasses. Distribution of other beef and small ruminant products is explained in the Appendix A.

Main structural vulnerabilities identified were similar as the one for cattle, with the addition that these companies are mainly dependant on export (not contributing to city supply).

### 3.3 Spatial maps

#### 3.3.1 Source of animals

**Fig. 8:**
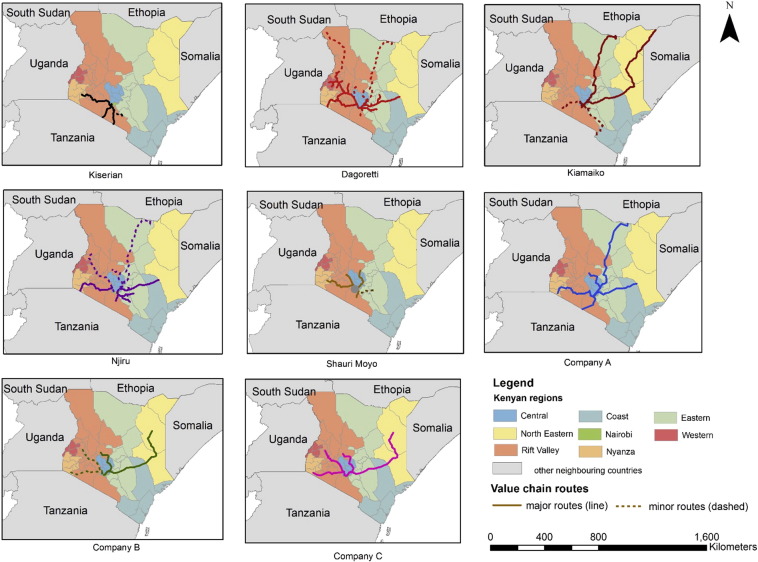
Geographical maps indicating source of ruminants for each of the markets supplying Nairobi.

Analysis of geographical supply routes resulted in each terminal market having a unique geographical pattern of influence based on the combination of main routes used (Fig. 8). Kiserian markets mainly reported to obtain most of their supply from the south of Kenya (all the way to Tanzania); Dagoretti markets from south-west and central-west Kenya (minor routes reach Uganda and Sudan); Njiru markets from south-west routes; Kiamaiko's small ruminants supply was obtained from East North Kenya (as far as Somalia and Ethiopia). Shauri Moyo market obtained almost half of its beef meat from Nairobi LTMs (Dagoretti, Kiserian and Njiru), up to 26% from Nairobi neighbouring counties (Machakos and Kajiado) and the rest form distant terminal markets. City market sourced 40% of beef meat from Shauri Moyo market, 25% from Limuru slaughterhouse, 10% from Dagoretti, 8% from Kiserian, 8% from Njiru and 5% from Kiambu. It sourced 90% of the sheep and goat meat from Kiamaiko, while the rest is sourced from Dagoretti (5%) and Kiserian (4%). The three large processing companies had a similar geographical pattern. The main route was reported to be the central-west routes (mainly Garissa market), used by the livestock traders. The supply of cattle from ranches originated from Laikipia.

#### 3.3.2 Destination of products

Markets reported to distribute the meat throughout Nairobi, but with higher influence near their location. However they also indicated to sell to the surrounding towns. For the LPCs the majority of beef (60–90%) was explained to be sold to the Nairobi market, while the rest is distributed to other areas in the country and large tourist hotels in the coast. Small ruminants from the large processing companies were reported to be exported to United Arab Emirates and some to Qatar, Oman, Saudi Arabia, Uganda, Southern Sudan, Angola and Rwanda.

Main structural vulnerabilities were: (1) north central and north east areas depending on Kiamaiko and Dagoretti's market and LPCs, and viceversa; (2) south rural areas depending on Kiserian and Njiru, and viceversa; (3) central and norths west rural areas depending on Dagoretti, and viceversa; (4) City market depending on Shauri Moyo.

### 3.4 Temporal maps

**Fig. 9:**
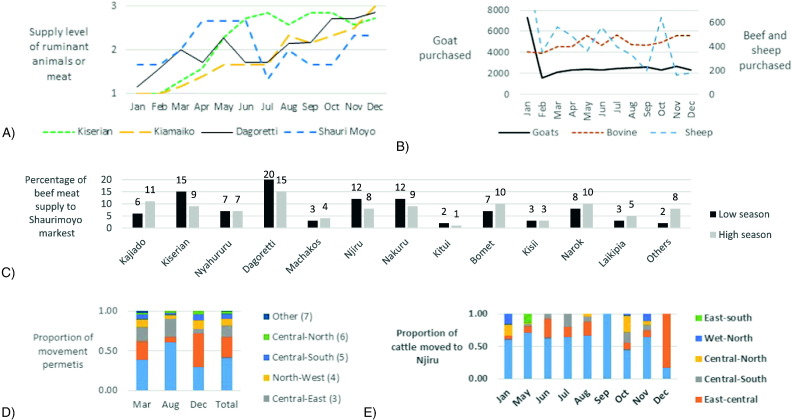
A) Trends of seasonality for LTMs and Shauri Moyo market. The graph shows the monthly mean supply scores for each market, where 1 = low supply, 2 = average supply and 3 = high supply; B) Number of beef, sheep and goat supply to a large processing company; C) Percentage of beef meat supply to Shauri Moyo market from different sources during the high and low season as reported by the meat inspectors; D) Proportion of cattle of animals moved to Dagoretti market in March, April and December 2012–2013; E) Proportion of routes used for beef for different months of the year (May 2013–Jan 2014) to Njiru market.

Seasonality was reported to depend on the dry and rainy seasons, and on festivities, such as Christmas. During the low season dominant markets increased their market share up to 12% for beef (in Shauri Moyo market) and 17% for small ruminant meat (in Kiamaiko market). Fig. 9 shows the temporal profiles for the different markets. Results from individual interviews showed an increasing demand pattern of meat in the year (Fig. 9a), with differing peaks for Shauri Moyo market (April–June) and LTMs (August–December). City market data showed December to be the highest month for supply and sales of sheep and goat meat. Further analysis on seasonality of Shauri Moyo market indicates that their supply from Nairobi LTMs increased during the low season, while their supply from distant terminal markets increased during the high season (Fig. 9c). For LTMS, temporal fluctuation of routes for Dagoretti and Njiru markets, as calculated from cattle movement permits, is shown in Fig. 8d and e. For Dagoretti, a total of 878 movement permits were obtained (250 for March, 247 for August and 381 for December). This represented a total of 17,087 animals moved in these three months (4665 in March 5183 in August and 7239 in December). Its analysis showed that Route 1 (South-West) doubles its supply to Dagoretti in August compare to March, while in December it declines to 29%. On the other hand, route 2 (Central-West), becomes the most important route in December (with 42% of supply), while in August its contribution is minimal (only a 6%). For Njiru market, in total 344 movement permits corresponding to movement of 7818 beef cattle in 10 month (January and May to November 2013), and 6 movement permits in December 2012 were obtained. Fig. 8e shows that the West-south route was the predominant one over the year for this market. Seasonality data obtained from one LPCs showed that the peaks for purchase of sheep were March, June and October. For goat, no evident peak was observed, except January which, also for sheep, was observed to be an exceptional month in 2014. For beef the peak were located in May, July, November and December.

## 4 Discussion

The mapping presented is unique in its kind, as it provides a level of detail on the diversity of red meat flows of a large fast-growing city not documented beforehand, and uses data from all livestock terminal markets in the city, the two major meat markets and the three largest processing companies. It helped to understand the complexity of the system flows and identify deficiencies and vulnerabilities associated to its structure. There are several potential applications of the results of this mapping analysis as illustrated throughout this discussion.

The diversity of chains and people operating in the LTMs and MMs, and the quantification of their flows, provide an understanding on the importance of different people in the control of flows. An important example is the different type of beef traders operating in the meat markets, with 80% being small traders; the identification of ‘walk-ins’ in high seasons, or the understanding that 12% of traders account for 50% of supply of meat in Dagoretti. These results were consistent with Onono et al. (2015), who showed the importance of large livestock traders in these markets, where about 60% of supply was controlled by 20% of traders. The mapping also allows to understand the dependency of different people to specific sources, products or other key stakeholders. Results indicate that LTMs and LPCs rely on independent livestock traders for 80% and 60% of their livestock supply, respectively, and that these depend on primary markets. In the literature, primary markets were reported to account for up to 90% of the supply of ruminants to Kiserian market (Mbiyu, 2015). These leads to a lack of traceability of animals, with inspectors, LPCs and meat traders not having any information or control on their initial source and their production management, and therefore being vulnerable to disease outbreaks. Brokers of meat and livestock were reported extensively in LTMs and MMs, and provide a linkage with retailers and between traders. These were reported to influence the setting of prices of animals and products in the markets. This influence has been highlighted in other studies, which described them to operate as ‘a cartel’ (Aklilu, 2002). Economic studies showed that the structure of the LTMs system, with numerous traders and brokers, favours benefits distribution to these while limiting the capacity of livestock holders to improve their production (Makokha et al., 2013). Policy makers aiming to make a change in the system may need to consider all the different chains and flows in the markets if effective interventions are to be implemented, especially those people accounting for a large proportion of the flows, while also protecting and regulating other people depending on minor chains.

Several key governance features can be derived from this investigation. The chains flowing through LTMs and MMs, normally known as informal markets (Kenya Market Trust, 2014), could be classified as ‘market value chains’ according to Gereffi's governance classification (Gereffi et al., 2005). These chains present a lack of standard grading of livestock and meat, corresponding with a lack of value addition on products. Standard products are therefore traded (such as raw meat or raw offals), which were simply codified, generally as high or low quality, but based on subjective perceptions and specifications. Pastoralists, traders and retailers associated to these markets have the capacity to produce livestock or products with little input from their buyers. As a consequence, as illustrated in the results, there are numerous independent stakeholders operating in the flow of products and these reported to worked with multiple partners. The mapping analysis showed however the main trend and destination of products in these markets. The cost of production for this market could be considered as low, compare to LPCs, and the cost of switching to a new supplier or buyer was also reported to be low by several people interviewed in these chains. This benefit these markets to supply to a large range of consumers, accounting for almost 90% of the Nairobi market. Especially, low income consumers ruminant meat supplied was reported to be mainly channelled through the MMs. It however represents an important barrier to entry to high end market and export opportunities. Several studies have highlighted the issue of lack of value addition and relate this to low economies of scale, lack of demand for value added products, lack of marketing strategies and technological/management constraints (Aklilu, 2002, Kenya Market Trust, 2014). However, despite these ‘market value chain’ characteristics, from a system point of view, it was shown that two specific markets (Shauri Moyo and Kiamaiko) were clearly dominant in the supply of beef and small ruminant meat. These markets were reported to be of importance because of their cheap meat prices. It is likely that the people in these markets have a key role on the governance of the system and the setting of prices. However, their large market share could make the system vulnerable to shocks if those markets were to be affected. This is of special relevance, as these markets have been the focus of important concerns, because of illegal activities and food safety risks (Achuka, 2013, Kiarie, 2014, Nairobi City Council Assembly, 2014, Ndonga, 2012). Market closure has been planned by the government for Kiamaiko, so to be replaced with new modern abattoirs situated elsewhere in Nairobi (Neema abattoir currently operational) (Nairobi City Council Assembly, 2014). Despite the availability of a new operational abattoir, Kaimaiko remains open, likely due to its importance in the system and social concerns. This study shows that future interventions and policies aiming to improve system efficiency and city food security and food safety will need to consider these markets and their economic and social importance if change is to be achieved. Closures and shock in these specific markets could have devastating consequences in food supply and livelihood of numerous people in the city, and should be the focus of debate between policy makers, food system and urban planners and private industry.

On the other hand, the chains flowing through LPCs could be classified as ‘modular value chains’ (Gereffi et al., 2005). Two out of the three LPCs had well stablished standard beef cattle and meat grading procedures, with people employed and specialized solely on this activity. They produce complex value added branded products which allow them to access higher end retailers, but also government institutions and low end markets with some less quality products (such as canned meat or beef sausages). These companies reported to use complex machinery and infrastructure (all declared to be ISO certified) and integrating processing and distribution activities. However, as consequence, these LPCs reported to have high cost of production, making their products of difficult access to average and low income consumers in Nairobi and to compete with LTM and MMs. Results of this study showed indeed that LPCs only represent a very small proportion of beef, sheep and goat market shares in the city, and with dependency on exports. However, within the high end market niche, cost of switching to another LPC is relatively low. It is important to note that this study aimed to map the system and that ongoing research focuses on governance, upgrading and distribution of benefits in the system, as required for the completion of a full value chain analysis and the identification of further inefficiencies and opportunities for public policy and private strategy. This mapping study represents the first step for this analysis and an essential framework to support future research on these areas.

Several important food safety risks and inefficiencies were identified from the system structure. The ‘disorganised’ system reported in LTMs generates accumulation of livestock in the markets holding grounds. As a consequence some animals were reported to stay for long periods of time in these areas and were circulated within the city for feeding and water, or to move them to another LTMs, representing a possible source of environment contamination and disease transmission. Long trekking of animals, identified to be associated to Kiserian and Dagoretti markets, represent another potential source of disease transmission. In addition, the fact that most LTMs operate without order make the clean area of abattoirs to function as market places, creating potential source of meat contamination. This problem was reported to be higher during festive season when large quantities of walk-ins also operate. An important feature of LTMs and MMs was also the management of low quality or ‘late’ carcasses, in occasions created due to abattoirs operating without orders and consumers' preference for “hot meat” (meat not store in fridge and recently slaughtered). This was identified to represent a source of meat to low end restaurants and butchers, and therefore to poor households. This meat could potentially be a source of pathogens, due to the prolonged exposure of carcasses to ambient temperature. It also indicates an important wastage of carcasses (either through trimming of carcasses, decrease in value or complete disposal) derived from the inefficiency of the system. In the case of Shauri Moyo market, long distances were reported to be travelled to transport meat without refrigeration, which could represent another important food safety problem. For LPCs, as also for LMTs and MMs, the lack of traceability and therefore lack of control of animal disease management on farms, represent an important gap for disease control. However, LPCs strategy to keep the animals in buffer zones was identified as an important practice to minimize the risk of disease animals reaching the abattoirs. Policies oriented at improving market facilities to control animal flows and to organise business transactions may improve system efficiency and reduce disease hazards in the systems. Improving standardisation of livestock and meat grading in LTMs and MMs, would potentially contribute to improve efficiency of the system and allow for adequate flow of information of animals and products to stakeholders and to generate market opportunities. Moreover, interventions aiming at better preserving these carcasses, for example through meat processing, such as beef sausage, while maintaining its availability to poor people should be explored. An example of successful approach was identified in the LPC B, which system is able to distribute large quantities of processed products (namely sausages) in informal settlements throughout a network of road-side vendors. However, nutrition-sensitive interventions in these systems should also consider the importance of ruminant offal, legs and head, as being the products most distributed to low class retailers, and thus to low income consumers. The results of this mapping study provide the basis for future research to investigate pathogen flows across the system, locate the risks and understand population exposure to these risks. Policy makers involved in disease and/or food safety control could use this framework to provide regulations or asses risk exposures from hazard occurring in the different chains.

Results from geographical and temporal mapping provided important information on sources and seasonal effects of the system. The results showed how livestock is moved from all over Kenya and neighbouring countries (Tanzania, South Sudan, Ethiopia and Somalia) to supply Nairobi market. The routes described for main markets were in accordance to previous studies (Alexovich et al., 2012, Muthee, 2006). The results obtained showed the influence of different production regions to the supply of different Nairobi markets. The type of livestock production system (pastoral, agro-pastoral and mixed farm) have also clear distribution in Kenya, and therefore Nairobi markets investigated can be influenced by these (Cecchi et al., 2010). This indicates that shocks in the production of a region in Kenya would create significant disruption in specific markets. Also, shocks in a specific market may have important impact on producers and traders of the regions depending on these markets, as Nairobi consumers represent an important market for these rural producers due to the demand size and high prices. In the event of a shock, policy makers may require to focus efforts on the key areas or markets that would be affected by these shock. Furthermore interventions aiming at improving pastoral production and household nutrition in specific areas of Kenya and Nairobi should consider the market destination and routes associated with them (e.g. interventions in Kiamaiko can have important economic/nutritional impact in northern east areas of Kenya and in specific informal settlements in Nairobi, such as Korogocho). The mapping analysis provides policy makers with the tool to understand where to target these type of interventions.

Analysis of temporal mapping for routes in Dagoretti showed how the supply contribution from different routes changes by season. The importance of south-west routes in the month of August (in the middle of the dry season) and the increased importance of central-east routes in March (beginning of the long rainy season) and December (high demand peak period and short rainy season), could indicate higher sensibility of south-west Kenya pastoralists to dry season. It was reported that these pastoralists prefer to keep their ruminants in March to allow them to grow during the rainy season, and to sell them in months such as August when animals are not able to grow due to scarce pasture. Pastoralists in central-west, less sensible to drought, might prefer to sell in March and December to benefit of higher prices. Seasonality results from overall market supply in the city indicate that the main markets such as Shauri Moyo and Dagoretti for beef and Kiamaiko for sheep and goats, are better able to obtain supply of livestock/meat to their market during low season, possible due to their higher diversity of sources and larger number of traders. Temporal trends observed from Shauri Moyo market indicates however their increase dependency of Nairobi LTMs for their supply during the low season, and therefore partly relying on these markets to obtained enough supply.

There are limitations that need to be considered when interpreting the results. Majority of the information is based on qualitative data or estimation of proportions obtained through focus group discussion or key informants. The lack of capacity of the project to interview a representative number of each people in the chain, lead to the need to obtain most of the estimations form key people in positions to understand overall patterns in markets, such as meat inspectors. Therefore, some estimations represent approximations based on perceptions and experiences. However, for this study researchers interviewed the different type of people in the market to allow for triangulation of some of the information and minimize errors. For example, information not revealed by some people, possibly due to lack of trust or illegal practices, such as movement livestock out from LTMs, was revealed by other people interviewed. In several cases, information was contrasted with other group of people to check on their validity. Final results were also presented to other key informants in the system to assess for errors and validate the results. In this process one important possible error was mentioned, which could not be corrected due to lack of agreement and validation. It was mentioned that Shauri Moyo market sourced a large proportion of beef meat from an abattoir situated in Machakos. However, market key informants were visited again and did not confirm this. It is also important to note that question formulated were designed to avoid leading answers and that presence of official (i.e. meat inspector or veterinary officers) or dominant people (i.e. abattoir owners) were avoided when possible to minimize respondent bias.

Lack of available data was also an important limitation in this study. For some markets, movement permits allowed for identification and quantification of sources of livestock. Unfortunately, for most markets these were not available. However, the results from this study show the potential of this data for analysis of geographical and temporal patterns, which could be used to understand and monitor system vulnerability to shocks in different areas and periods. Currently, this information is not being used, as its only purpose is to ensure that animals are moved with authorization. Further limitations are the absence in this study of other abattoirs outside Nairobi (but which contribution to Nairobi supply was reported minimal by informants in the ministry headquarter) and absence of other large companies such as Alpha Fine food (distributing 5200 cattle carcasses and 26,000 small ruminant carcasses per year in Kenya), which should be considered in future studies describing the system (Kenya Market Trust, 2014).Nonetheless, this study is based on two years of extensive and complex data collection in the major markets and companies, which combined with the diversity of methods used, the triangulation of information and validation of results, it allow for an accurate and detail picture of the ruminant food system.

## 5 Conclusion

Three important segments in the system were identified, the LTMs, MMs and LPCs. From these LTMs and MMs supplied to the large majority of the city and operate as a ‘market value chain’, but with two markets (Shauri-Moyo and Kiamaiko) controlling most of the supply. Analysis of people and product profiles identified the large diversity of flows, people and products in these markets, and highlights the importance of livestock traders in LTMs, and of small meat traders in Shauri Moyo market. Low end retailers were identified to source meat and offals form long chains (passing through MMs), and to access low quality and potentially degraded products. LPCs operate as a ‘modular value chain’, but with important dependence on livestock traders and primary markets for the supply of ruminants and on export markets for the distribution of sheep and goat products. However, one LPC presented an efficient business model in the distribution of low end products (e.g. sausages) to average and low income households in Nairobi. It also highlight key structural deficiencies in LTMs and MMs, such as lack of value addition or a disorganised system with inefficient traceability, accumulation of livestock for long periods in the markets, promotion of extensive broker activity, abattoirs operating as market place, wastage of carcasses and others, many representing potential disease transmission hazards and limitations to access high-end and export market. Results of the geographical and temporal profiles provides an understanding on system vulnerability to shocks associated to specific regions or markets. This study provides the framework for interventions studies and policies aiming to improve the efficiency of the system, and shows a methodological approach for mapping of other systems. The framework used represents an important backbone to overlay research on chain governance, barriers to entry, food safety risk practices and pathogen flows needed for a full understanding of the functionality of the system. Furthermore, the results have the potential to be used as a stepping stone for quantitative value chain simulations models as described by Hamza et al. (2014), Lie and Rich (2016), Naziri et al. (2015) and which would be useful to predict the impact of shocks to the systems described.

## Ethical approval

Ethical approval for this study was obtained from the ILRI Institutional Research Ethics Committee (ILRI IREC) (project reference: ILRI-IREC2014-04/1). ILRI IREC is accredited by the National Commission for Science, Technology and Innovation (NACOSTI) in Kenya. Furthermore, ethical approval from the Royal Veterinary College ethical committee was also obtained (project reference: URN 2013 0084H).

## Conflict of interest

None.

## Figures and Tables

**Table 2:** Grading specification for beef cattle and beef meat for one large processing company.

Type of grade	Type of animals	Conformation	Fat	Other
Prime	Steer and maiden heifers bull with milk teeth	Steer & bulls of 180 to 310 kg heifers 160 to 310 kg 18–30 months	15 mm maximum white or creamy colour firm and evenly distributed	No or slight evidence of cartilage ossification of the thoracic vertebrae good conformation
Choice	Steers and heifers with maximum six permanent incisors	Steer & bulls of 180 to 320 kg heifers 160 to 320 kg 24–42 months	20 mm maximum firm and evenly distributed	Hindquarters free from blemishes
Fair average quality (FAQ)	Steers, bulls, heifers and cows	140 to 340 kg	20 mm maximum fairly distributed and not excessively yellow or oily	No extensive and penetrating blemishes
Standard	All	No limit	Some covering of fat bull with good fat cover	No extensive and penetrating blemishes
Commercial	All	No limit	No limit	Severely blemished
Manufacturing	All	No limit	No limit	Extremely and extended blemished > 6 measles cysts

**Table 1:** Focus groups discussions (FGDs) and key informant interviews (KIIs) done for this study.

Node	FGDs (participants)	KIIs (No.)
Kiserian	7 FGD with pastoralists (4); livestock traders (13); meat traders (6); livestock transporters (6); meat transporters (5); abattoir owner/managers (4); abattoir butchers (8)	Meat inspectors (2); abattoir owner (1)
Dagoretti	10 FGD with livestock traders (5); meat traders (6); livestock transporters (4); meat transporters (5); abattoir owner/managers (4); abattoir workers (6); fillet traders (6); skin traders (7); veterinary officers and meat inspectors (7); offal traders (5)	
Kiamaiko	7FGD with livestock traders (20); meat traders (8); meat transporters (5); abattoir owner/managers (4); skin traders (5); meat inspectors (4); flayer and offal traders (9)	Representative of livestock transporters (1)
Njiru	–	Meat inspectors (2), livestock trader (2), livestock transporter (1)
Shauri Moyo	6 FGD with meat retailers (2); meat traders (8); meat transporters (5); market managers (2); meat inspectors (4); city council representatives (2)	Meat inspector (1)
City market		Meat inspector (1), city council (1), meat retailer (1)
Large processing companies	1 FGD with large company 1: veterinary and general manager (2)	Large company 2: marketing, supply, production and veterinary managers (4); large company 3: supply, marketing and quality managers (2)

## Footnotes

1 Percentages do not sum 100% because animals and meat can move through more than one market as shown in Fig. 2.

2 0.0116 USD/KES [31/12/2013].
